# Memoir on the Radical Cure of Stuttering by a Surgical Operation

**Published:** 1841-07

**Authors:** 


ART. XVII.

1. *Memoir on the Radical Cure of Stuttering by a Surgical Operation.* By J. F. DIEFFENBACH. Translated from the German by JOSEPH TRAVERS.—*London,* 1841. 8vo, pp. 27.

2. *Du Bégaiement, &c.* Par le Dr. C. PHILLIPS.—*Paris,* 1841. pp. 63. *On Stammering, &c.* By Dr. C. PHILLIPS.

3. *Stammering and other Imperfections of Speech treated by Surgical Operations on the Throat.* By JAMES YEARSLEY, M.R.C.S.—*London,* 1841. 8vo, pp. 46.

4. *On Stammering and Squinting, and on the Methods for their removal.* By EDWIN LEE, M.R.C.S.—*London,* 1841. 8vo, pp. 88.

THE sanguinary operations which have been recently devised and executed, with the view of curing stammering, are one of the greatest outrages upon modern surgery. Although some of them had their origin in legitimate motives, most, we fear, serve but to show what ruthless expedients will be occasionally resorted to for the purpose of acquiring professional fame, however short-lived, and to what extent the ignorant and credulous will become a prey to craft and subtlety. If our indignation was awakened at the barbarous cruelties practised upon dumb animals for the sake of elucidating the truths of physiology, how much more ought it to be when we consider the multitude of our fellow-beings who have suffered themselves to be maimed and mutilated at the instigation of individuals more remarkable for their reckless use of the knife than for the soundness of their medical science!

Any one who calmly and dispassionately studies the phenomena of stammering will be convinced that it is not referrible to the defect of any single part, but, as Sir Charles Bell has pointed out,* to imperfection in the power of combination of the various parts which are essential to produce the simplest articulate sound, and depending on some derangement of the nervous system. Indeed, it is well ascertained that persons who have stammered in the highest degree, have been remarkable for the perfect integrity of conformation and structure of all the organs of voice and speech; while others who have laboured under a faulty or diseased condition of these organs have preserved their articulation unimpaired. There are even several well-authenticated cases upon record of individuals who have been able to discourse fluently, notwithstanding the absence of the tongue.†

In corroboration of the nervous and spasmodic view of the affection in question, we would merely allude to the well-known influence exercised by mental emotions in either aggravating or removing for a time defective utterance, and to the established fact of the efficacy of judicious mental and moral training in accomplishing generally a perfect cure. Every one is familiar with the classical instance of Demosthenes, who, although he stuttered while a youth, became the most distinguished orator of antiquity.

The work of Professor Dieffenbach is entitled to consideration from the eminent position its author has acquired as an operative surgeon. How far his reputation will be enhanced by its publication, so injudi-

* On the Organs of the Human Voice, Phil. Trans. 1832.

† Jussieu in Mém. de l'Académie Royale des Sciences, 1718, and Phil. Trans. 1742.

ciously premature, is a most doubtful matter. The translation by Mr. Joseph Travers is a very creditable performance. The author informs us that after his attention had been directed for a considerable time to the painful condition of the stutterer, he came to the conclusion that the difficulty of pronunciation emanated from the larynx, was communicated to the tongue, and that in order to subdue it the sole means was division of the nervous and muscular agents at the superior part of the base of the last-named organ. He was led to frame the above hypothesis from analogy, that is to say, from witnessing the almost invariably favorable result which followed the division of the muscles of the eye, in what is called nystagmus bulbi.

However ingenious and plausible the above views may appear, we do not believe they will stand the scrutiny of physiological enquiry. In nystagmus bulbi the muscles of the eye are alone affected, and by cutting them across we may no doubt modify their irregular action; but we cannot conceive how a transverse section of the whole muscular substance of the root of the tongue can induce any permanent change on an apparatus so complicated as that required for the production of speech. It is ascertained that the compression of the thorax, the adjustment of the larynx and glottis, the motions of the lips and tongue, and the actions of the pharynx and palate, must all consent before a word be uttered. Now we have been given to understand that experience has demonstrated the fallacy of the theory; for out of thirty-two cases in which the professor has operated, not more than three have derived anything beyond temporary benefit. The marvellous effects stated to have followed the operation in the first instance, are in all likelihood due to the sudden shock given to the nervous system in highly-impressible subjects. We have been told of a well-authenticated case of a German officer who was cured of stammering through a sword-wound on the face, received in a duel.

The Berlin professor has given trial to the three following methods of operating; they have equally for their object total division of the root of the tongue: "I. The transverse horizontal division of the root of the tongue. II. The subcutaneous transverse division, in which the mucous covering of the tongue is left inviolate. III. The horizontal division, with excision of a wedge-shaped portion." (p. 9.) The last method is the one upon which he places most reliance, "as here some shortening of the tongue must necessarily ensue; and forasmuch as the base of the wedge-shaped slice is made from the dorsum of the tongue, elevation of the tip must take place. This method, then, mechanically assists that organ to assume the position insisted on by those teachers who have been most successful in ameliorating this defect." (p. 10.)

The mode of performing the operation, which was done for the first time on the 7th of January last, is thus described:

"The boy sat with his head leaned against the breast of an assistant; the tongue being protruded as far as possible, was grasped on its anterior half with the forceps of Müzeux, being thus compressed laterally, and drawn forward by one assistant. The gentleman against whose breast the boy's head rested retracted the angles of the mouth with a pair of blunt hooks. Grasping now the tongue as near to its root as possible, between the thumb and forefinger of the left hand, I passed the bistoury through it, and divided it completely from below upwards; a strong ligature, passed through the posterior edge of the wound, served to fix it temporarily, and prevent too great a strain upon the slender band which alone connected the mass of the tongue to it; the anterior lip of the incision was now grasped, and laterally compressed between the modified hare-lip forceps and a wedge-shaped slice excised out of the whole thickness of the tongue. It will be found more convenient to make this second incision from above downward, and with a small straight knife. The posterior edge of the wound was now, by means of the before-mentioned ligature and a sharp double hook, drawn so far forward that the needles with the ligatures could be conveniently passed through it; six strong sutures served to bring the edges of the wound together and to restrain the hemorrhage. To effect the latter object, they must include the whole depth of the wound within their loop. That the hemorrhage was considerable may be imagined from the nature of the operation, which should not be attempted by all persons indiscriminately." (p. 13.)

For the simple transverso-horizontal division of the root of the tongue, the following procedure was adopted:

"The tongue being fixed as in the foregoing case, and its root cut transversely through, six strong sutures were applied, which brought the wound effectually together. As in the preceding cases, the bleeding was considerable at the time, but completely stopped by the application of the sutures." (p. 20.)

Subjoined is the mode of effecting "subcutaneous division of the root of the tongue:"

"The tongue being drawn as much forward as possible, I pushed a curved bistoury through it, as near its root as I could, and cut through its whole muscular thickness, leaving the mucous membrane inviolate; on the withdrawal of the knife the opening appeared only of the breadth of the blade. The substance of the organ was so completely cut through that a slight additional pull with the forceps would probably have torn it off. The blood streamed from the apertures made by the knife as vehemently as from a large artery, whilst at the same time the cavity of the wound became exceedingly distended with the rapidly-flowing blood. This cavity I sought to diminish by introducing a strong suture from behind forwards through the tongue, and with other sutures I closed the openings made by the bistoury on each side." (p. 22.)

The results of the last-mentioned methods of operating were by no means satisfactory.

The few concluding pages contain some details, *in extenuation*, touching the treatment of wounds of the tongue, the removal of the sutures, and the phenomena of cicatrization. The following summary of "contingencies rationally to be feared, and which must be carefully weighed beforehand," more especially when coupled with the fact of the very doubtful success of the operation, will, we think, deter any rational surgeon, albeit endowed with "the temperament of an operator," from again repeating the operation in this country.* "The extent and importance of the operation, the possible danger to life, or loss of the tongue, either through the want of skill in the assistants, who may tear it off when so nearly separated, or through mortification or ulceration of its connecting isthmus." (p. 26.)

The portion of M. Phillips' *brochure* devoted to the subject of stammering occupies twenty-two pages, whereof nearly nine are filled with

* It has been twice performed in London, first by Dr. Franz, afterwards by Mr. Bennet Lucas. We have been informed that the result of Dr. Franz's operation, which was skilfully done in strict accordance to the precepts of Dieffenbach, has proved a failure. The effects of hemorrhage in the first instance were most alarming. For an account of the latter case, the reader is referred to the Provincial Medical and Surgical Journal for May, 1841.

extracts from M. Amussat's communications to the Academies of Sciences and Medicine, and about three with the details of Dieffenbach's plans of operation, leaving a balance of ten for the observations of the author, which evince too controversial a spirit to interest our readers. He modestly tells us, in concluding, "*Cette petite esquisse historique est aussi complète que possible*"! (p. 63.)

M. Amussat, assuming that the cause of stammering resides most frequently in faulty conformation or undue contraction of the genio-glossi muscles, together with shortening, deviation, or deformity of the tongue, recommends and practises for its relief the section of those muscles near their attachment to the tubercle on the inside of the symphysis of the chin. The number of subjects operated upon by him up to the middle of March last were thirty-three, whereof three were females. The results obtained, he assures us, were quite satisfactory; the cessation of the stuttering supervening sometimes directly after the cut, sometimes not before the lapse of a few days. In a certain number the defect altogether disappeared; in others again there was only a slight improvement. In six cases the mere division of the frænum, together with the mucous and fibrous textures lying upon the muscles, sufficed to effect a cure. The following procedure, suggested by M. Bonnet, (Gazette des Hôpitaux, Avril, 1841,) which is but a modification of that of MM. Amussat and Phillips, appears to be the safest mode of accomplishing the section in question; since considerable hemorrhage and inflammation have occasionally followed the other methods described.* According to M. Bonnet, an opening is to be made beneath the chin. You thus avoid all injury of the mucous membrane of the mouth, all effusion of blood into that cavity, and while you completely sever the genio-glossi muscles, you do not interfere with the genio-hyoidean portion, and at the same time obtain all the advantages resulting from subcutaneous incisions, namely, immediate cicatrization of the external wound, without any risk of suppurative inflammation. He directs a puncture to be made along the median line three or four *centimetres* behind the chin. Through this puncture he introduces a blunt *tenotome*, making it penetrate from below upwards, and a little from behind forwards, its cutting edge being turned towards the jaw. When it has arrived just beneath the mucous membrane of the mouth, as is ascertained by passing the left index finger into that cavity, he feels for the little bony tubercle and cuts away from it both right and left, keeping the edge of the instrument always towards the lower jaw. By this precaution you secure the section of the genio-glossi muscles at their bony insertion. The *tenotome* is not withdrawn until the finger again introduced into the mouth enables the operator to feel that its extremity is in contact with the mucous membrane, that the fibrous texture connecting that membrane with the jaw is wholly severed in the mesial line, as recommended by M. Amussat, and that there are no longer any muscular fibres adherent to the above-mentioned tubercle. In this way, the genio-glossi muscles being cut across in their aponeurotic portion, we do not interfere with the layer of cellular texture placed beside them, nor incur any risk of wounding the lingual arteries. There is very little blood lost. The patient can generally speak and move about on the next day, complaining only of a little stiffness in the movements of the mouth.

* Revue Médicale, Mars, 1841, p. 460. Phillips, p. 46.

This operation, unluckily for its author, has failed in other hands. M. Guersent jun. states (Gazette des Hôpitaux, Avril, 1841,) that out of nine cases in which he had tried it, one only was anywise benefited—an issue far from encouraging. Besides, we cannot tell what may be the ulterior consequences of severing these muscles from their bony attachments. We know that they offer the sole resistance to the movements of the tongue backwards; and we also know that in certain operations about the jaw, imminent danger of suffocation has ensued from the tongue being reversed upon the epiglottis and larynx where these had been cut across. It is true that the preservation of the mucous membrane may tend to counteract so grave an accident; still we are of opinion, that from all the evidence we have been able to collect, there is no good ground to sanction the above operation being performed under ordinary circumstances.

Mr. Yearsley informs us in his pamphlet* that nearly 200 persons, affected with every variety and degree of stammering, have passed under his notice. Of these a large proportion presented enlargement of the uvula and tonsils, while many were short-breathed, exhibiting the narrowing of the chest termed chicken-breasted. The only novelty to which he lays claim "is the promulgation of the facts, that, *in the great majority of stammerers, the tonsils and uvula are in a diseased state, and may be removed with advantage, and that these operations may in particular be applied to the relief of stammering and imperfect speech.*" (p. 11.)

According to him, spasmodic closure of the fauces from obliteration of the isthmus, is the chief if not the sole cause of stuttering. The theory of *nervousness* he wholly repudiates, believing it "to be most erroneous, and applicable solely to a few cases where the stammer leaves the patient sometimes for several days together, and then suddenly recurs." (p. 24.) In short, the very pith and marrow of his doctrine may be enunciated in the words of the Mantuan poet, "vox faucibus hæsit."

The most beneficial effects, he assures us, occurred in the most aggravated cases, and those longest operated upon were most advanced to a perfect cure. He excises the tonsils with a knife, and amputates the uvula with a pair of curved scissors. He observes that no accident of any consequence has happened in the performance of these operations, and in only one instance was the bleeding of sufficient importance to require any interference.†

A formidable and suspicious-looking array of cases is annexed in the

* The pamphlet is dedicated to the members of the Westminster Medical Society, "in return for the honour they did the author in inviting him, though not a member, to read a paper to them on the subject of his discovery." This would seem to be a misstatement; for it is declared in a report of a meeting of the above society of the 17th of April last, contained in the Lancet of the 1st of May, that "On referring to the minutes, it was found that Mr. Yearsley had *not* been *invited*, but by an act of courtesy in the society, had been *permitted*, although merely a visitor, to read the paper in question."

† As monkeys are the only animals which possess a uvula exactly resembling that of man, we would suggest to Mr. Yearsley a trial of its excision in them, as a curious matter for speculative investigation. Perhaps it might improve their chattering: who knows but in his hands it might make them talk? The doctors of the Sorbonne were puzzled why apes should not speak, seeing that they possessed all the organs of voice.

form of an appendix, several of which are reported as cured, others as considerably improved, while others remain unchanged. Now, we considered ourselves bound in common justice to Mr. Yearsley to examine some of the individuals above referred to, and for that purpose perambulated various "back slums" of the metropolis, till we arrived at their places of residence. The result of our inquiry, we regret to say, has been truly unsatisfactory. Indeed, we are thoroughly convinced that that gentleman has been most grossly and wantonly imposed upon by the crafty tribe of stutterers; for in some of the reputed cures we were told that the patients stammered as badly, or, if anything, worse, than before the operation. That the diseased condition of the tonsils and uvula, upon which the author lays such stress, may occasionally coincide with stammering, we readily admit; but that it is to be viewed as the cause of that affection, we utterly deny. Every surgeon in extensive practice has met with abundant instances of hypertrophied tonsils unattended with stammering. Nor is amputation of the uvula for impaired utterance a new thing; under that head Mr. Cooper distinctly recommends it in his Surgical Dictionary, when from enlargement "it becomes troublesome in deglutition and speaking." In conclusion, the expressions of "instant benefit" and "immediate relief," which we find pervading many of Mr. Yearsley's reports of cases, induce us to believe that the temporary good which has followed his operations is to be ascribed to nervous shock, and to nothing else. We have merely to add our entire conviction, that neither cutting a slice out of the root of the tongue, nor dividing the muscular bands which attach it to the jaw, nor "cutting the throat," can produce a sure and permanent cure of stammering.

Mr. Lee's slender volume reached us when the foregoing article was already in type. After some prefatory remarks on the voice and speech, the author reproduces the various opinions of Malbouche, Serres, Rullier, Magendie, and Colombat on the theory and cure of stammering, with judicious comments upon each. He then proceeds to detail very fully the different operative methods to which we have above adverted, and which, we trust, will ere long become obsolete in the practice of surgery. Mr. Lee evidently inclines to the division of the genio-glossi muscles as being the operation "in which the success has been the most permanent in bad cases of stammering." (p. 56.) He has, moreover, had the good fortune to see one "lucky hit" of Mr. Yearsley's, where relief from cutting the tonsils and uvula has continued to the present time (two months from the operation), and where the patient "can now speak with facility and without stammering." (p. 51.)

While we heartily concur in the conclusions Mr. Lee has deduced as to the nervous nature of the affection in the majority of cases, we take leave to differ from those which he has drawn as to the surgical treatment. We can, however, recommend his work to such of our readers as are desirous of making themselves master of this new subject, as by much the completest and best yet published on it in this country.

